# Understanding Discordance between In Vitro Dissolution, Local Gut and Systemic Bioequivalence of Budesonide in Healthy and Crohn’s Disease Patients through PBPK Modeling

**DOI:** 10.3390/pharmaceutics15092237

**Published:** 2023-08-30

**Authors:** Chunyan Han, Tiancheng Sun, Siri Kalyan Chirumamilla, Frederic Y. Bois, Mandy Xu, Amin Rostami-Hodjegan

**Affiliations:** 1 Centre for Applied Pharmacokinetic Research (CAPKR), University of Manchester, Manchester M13 9PL, UK; 2 Pharmaron Inc., Beijing 100176, China; tiancheng.sun@pharmaron.com (T.S.); mandy.xu@pharmaron.com (M.X.); 3 Simcyp Division, Certara, Sheffield S1 2BJ, UK; sirikalyan.chirumamilla@certara.com (S.K.C.); frederic.bois@certara.com (F.Y.B.)

**Keywords:** local bioequivalence, local BE, *f*_2_, virtual bioequivalence, gut-wall exposure, Entocort EC

## Abstract

The most common method for establishing bioequivalence (BE) is to demonstrate similarity of concentration–time profiles in the systemic circulation, as a surrogate to the site of action. However, similarity of profiles from two formulations in the systemic circulation does not imply similarity in the gastrointestinal tract (GIT) nor local BE. We have explored the concordance of BE conclusions for a set of hypothetical formulations based on budesonide concentration profiles in various segments of gut vs. those in systemic circulation using virtual trials powered by physiologically based pharmacokinetic (PBPK) models. The impact of Crohn’s disease on the BE conclusions was explored by changing physiological and biological GIT attributes. Substantial ‘discordance’ between local and systemic outcomes of VBE was observed. Upper GIT segments were much more sensitive to formulation changes than systemic circulation, where the latter led to false conclusions for BE. The ileum and colon showed a lower frequency of discordance. In the case of Crohn’s disease, a product-specific similarity factor might be needed for products such as Entocort^®^ EC to ensure local BE. Our results are specific to budesonide, but we demonstrate potential discordances between the local gut vs. systemic BE for the first time.

## 1. Introduction

The lifecycle management of drugs involves conduct of bioequivalence (BE) studies throughout various phases of drug development, necessitated by unforeseen factors such as changes in formulation or manufacturing process. These BE studies offer the possibility of comparing clinical outcomes by ensuring the similarity of the essential data for products going through various phases of development. Since the initial publication of the bioequivalence regulation by FDA in 1977, bioequivalence standards have undergone substantial evolution, incorporating increasing levels of complexity in drug discovery. However, pharmacokinetic endpoint studies remain the most commonly employed approach due to their sensitivity and repeatability, in contrast to pharmacodynamics or clinical endpoint studies [1]. The underlying assumption is that systemic exposure is directly linked to the therapeutic effect. When two pharmaceutically equivalent drug products (e.g., two formulations of the drug or two drug products) are demonstrated to be bioequivalent, it is inferred that their active ingredient(s) are absorbed at the same rate and extent, generating comparable safety and efficacy profiles when administered to patients under the specified conditions in the product labeling. Consequently, these two drug products are deemed therapeutically equivalent and can be interchanged without restrictions [2].

For locally acting gastrointestinal (GI) products, unlike other drugs, plasma concentration is downstream from the site of clinical effect, resulting in a disparity between the pharmacokinetic (PK) profile and effectiveness. After administration, the active drug ingredient would be released from the drug product, undergoes dissolution in the GI tract, and subsequently becomes available at the site of action for therapeutic purposes. The rate and extent of drug presentation at the site of action are governed by dissolution and the transit along the GI tract rather than plasma concentration [3]. In the case of such drugs, in vivo dissolution testing plays a critical role in identifying potential formulation difference between generic and reference products of GI locally acting drug products, assuming the formulation has no impact on the GIT itself, whereas plasma concentration actually disconnects from the action site drug concentration. Any changes in intestinal microbiota, absorption rate and the expression level of intestinal transporters, intestinal metabolism, hepatic metabolism and plasma protein caused by interindividual differences or patients’ disease state could bias the detected plasma concentration profiles and widen the gap between local GI concentration and in vivo PK profiles [3].

Considering the polarity between GI local concentration and systemic PK profile of GI products, developing physiologically relevant dissolution methods and the high cost of clinical endpoint BE studies pose significant challenges. In this context, physiologically based pharmacokinetic (PBPK) models emerge as an ideal investigative tool for exploring the correlation between dissolution in the GI tract and plasma concentration profiles. These in silico PBPK models can integrate existing knowledge of physiological parameters (and population distribution) and drug properties that influence oral drug absorption together, which allows theoretical investigation into the interplay between the system and the drug properties, shedding light on the main driving forces of drug absorption, transport and metabolism [4]. Verified PBPK models are also capable of predicting potential pharmacokinetics difference between test and reference drugs in virtual populations and thus assess the BE risks by virtual bioequivalence simulations [5].

In this study, the controlled-release (CR) formulation of budesonide, known as Entocort^®^ EC, was utilized as an illustrative example to compare local and systemic PK profiles in both healthy volunteers and Crohn’s disease (CD) patients. There are many different dosage forms of budesonide on the market today, i.g., nasal spray, rectal tablet, oral capsule, etc. [6]. Oral budesonide serves as the first-line therapy for inducing remission in mild to moderate CD patients [7]. It is a corticosteroid drug with high potency while experiencing limited systemic exposure due to extensive first-pass metabolism [8]. The permeability of budesonide is high (BCS class II), and if the compound is dosed in the form of micronized solid, it could dissolve quickly and be absorbed mainly in the upper GI sections [9]. The approved product Entocort^®^ EC is an orange/white-colored gelatin capsule containing 3 mg of budesonide in the form of small pellets. Each pellet consists of multiple layers, including an enteric coating on the exterior that initiates dissolution at a pH > 5.5 after the budesonide pellets enter the duodenum, and under the coating an ethylcellulose polymer–budesonide layer that can control the rate of budesonide release. This multiple-unit formulation can combine the favorable pharmacological properties of budesonide (high potency and extensive first-pass metabolism) with localized, time-dependent release targeting the ileum and ascending colon, the most common sites of inflammation in patients with Crohn’s disease.

Despite the extensive first-pass metabolism and low bioavailability of budesonide, it is feasible to accurately quantify its plasma concentration following the oral administration of Entocort^®^ EC capsules. Abundant clinical data collected with different dosage of Entocort^®^ EC capsule in a healthy population and CD patients are available in publications. Additionally, literature reports provide information on the dissolution profile of Entocort^®^ EC at fed- and fasted-state biorelevant buffer for healthy volunteer and CD patients at different disease levels. These resources render Entocort^®^ EC an ideal model drug/formulation for investigation in the current study.

In this study, PBPK models were built for Entocort^®^ EC in both healthy subjects and in Crohn’s disease populations. Local GI tract exposures in eight segments from duodenum to colon and two layers (lumen and enterocyte) in each segment were simulated for virtual generic formulations and Entocort^®^ EC. Local bioequivalence was calculated and compared with systemic bioequivalence. For each virtual simulation, correlation between *f*_2_ value, local bioequivalence and systematic bioequivalence was examined. Validity of *f*_2_ value to indicate local or systematic BE, or adequacy of systemic BE to demonstrate local BE was analyzed for all virtual formulations. Suitability of incorporating healthy volunteers in BE studies for formulations used in treating GI tract diseases was investigated. Through this current study, we simulated various scenarios representing actual BE studies, trying to formulate some guiding principles regarding the most appropriate methods for BE studies of drugs acting locally in GIT.

## 2. Materials and Methods

### 2.1. Workflow

**Figure 1:**
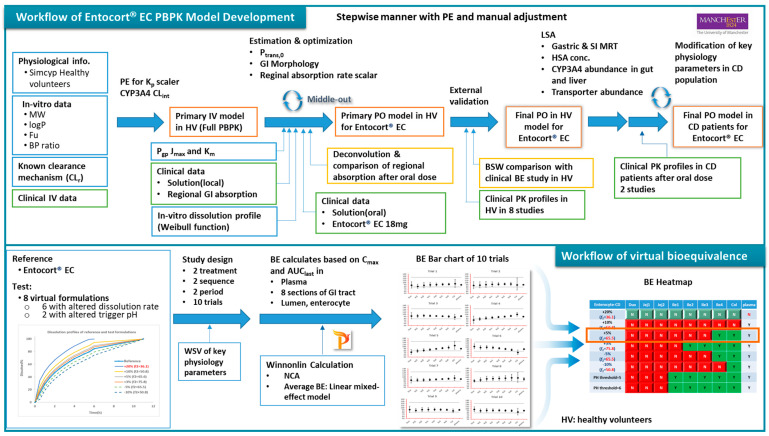
Workflow of the PBPK model and virtual bioequivalence in this study. PE: parameter estimation; MW: molecular weight; f_u_: unbound fraction; BP: blood plasma; IV: intravenous; Pgp: P-glycoprotein; PO: by mouth; LSA: local sensitivity analysis; SI: small intestine; HSA: human serum albumin; BE: bioequivalence; NCA: noncompartmental analysis; HV: healthy volunteer; CD: Crohn’s disease; BP: blood plasma, PPB: plasma.

The workflow of the study is as depicted in Figure 1. PPBK models for budesonide and Entocort^®^ EC were developed and validated against clinical PK data in HV and CD patients. Then, virtual bioequivalence studies were conducted with appropriate within-subject variances so that the results are closer to actual situations. Based on BE results, bar charts for 10 trials were generated and analyzed, and BE heatmaps were depicted.

### 2.2. Software

The Simcyp PBPK Simulator (Version 22 Release 1; Certara UK Limited, Sheffield, UK) was used to build the model for Entocort^®^ EC formulation in healthy subjects and Crohn’s disease patients. VBE module embedded in the simulator was used in simulations of virtual bioequivalence between Entocort^®^ EC and virtual generic formulations in eight sections of local GI tracts along with plasma in both populations. Clinical plasma concentration–time data from the literature were digitized with Digit (version 1.0.4, Simulation Plus). Noncompartmental analysis and bioequivalence analysis were performed using Phoenix WinNonlin 8.3 (Certara L.P., Princeton, NJ, USA).

### 2.3. Data Package Used in Modeling

Physicochemical, in vitro ADME parameters and clinical PK data collected from health volunteers and CD patients are available from literature sources. Budesonide clinical PK profiles in 10 clinical trials conducted with injections, solutions (orally and GI locally dosed) and Entocort^®^ EC capsules in healthy subjects and in two studies conducted with Entocort^®^ EC in CD patients were collected and used in the model development and validation. The summary of basic subject demographics information of these clinical trials is presented in Table 1. Since individual data were not provided in these papers, the mean concentrations from each trial were extracted instead. Clinical studies 1–4 were used in stepwise model building and parameter fitting. Others were used for external validation. For each simulation, the trial design was adapted to match the dose, age range and proportion of females in the reported clinical study under preprandial state. The number of subjects in each trial was set as 100, following the default setting in Simcyp.

### 2.4. Model Development and Validation

#### 2.4.1. Physicochemical Data

Budesonide is a moderately lipophilic (logP_o:w_ 2.62) neutral small molecule compound. Physicochemical and binding data were collected from Effinger’s paper [22]. The plasma protein binding was assigned to HSA and the K_D_ was calculated by Simcyp.

#### 2.4.2. Distribution

The full PBPK model was selected as the PK model. The Rodgers and Rowland equations (Method 2) were used to calculate tissue-specific K_p_ values and the volume of distribution. The K_p_ scalar was adjusted manually to 1.065 so that predicted K_p_ values for all tissues could be scaled and the predicted V_ss_ could resemble the reported volume of distribution in clinical PK study with IV dose, which is 2.69 L/kg [10]. After incorporation of clearance, the fitted IV plasma concentration showed overestimation of exposure (underestimation of IV plasma concentration profile). The K_p_ scalar was then adjusted to 0.8 to better fit the observed PK profiles.

#### 2.4.3. Metabolism

Numerous studies have reported that budesonide undergoes extensive first-pass metabolism, primarily mediated by the enzyme CYP3A4. Additionally, there is minor renal clearance involved in the elimination process. The renal clearance was calculated by Equation (1), where f_u_ was equal to 0.15, and the glomerular filtration rate (GFR) of the population representative (a 24-year-old Caucasian male), 172.42 mL/min/1.73 m^2^, was used in the calculation. Consequently, *CL_R_*, the typical renal clearance of budesonide for a 20–30-year-old healthy male, was calculated to be 1.55 L/h. Biliary clearance and additional systemic clearance were set to be 0 since no biliary excretion or other elimination route was reported for budesonide.

(1) $$CL_{R}=f_{u}\times GFR$$

Regarding to metabolic clearance, CYP3A4 was added to Enzyme Kinetics pane as the only metabolic pathway. Recombinant was selected as the source of kinetic data, and the intrinsic clearance (CL_int_) mediated by CYP3A4 was estimated by fitting the IV plasma concentration profile. A CL_int_ of 4.1 μL/min/pmol of isoform was estimated by PE function.

#### 2.4.4. Absorption

Multilayer gut wall within the ADAM (M-ADAM) model was used to simulate the absorption of Entocort^®^ EC. The model parameters were adjusted to mimic the behavior of the time-dependent release and absorption of the enteric-coated formulation along the intestinal tract.

1. Permeability

Apical P_trans,0_ was calculated by method 2 in Simcyp using Equation (2), where *a* = 2.36 × 10^−6^ and *b* = 1.1. The estimated Apical *P_trans,_*_0_ of budesonide was 1798.5 × 10^−6^ cm/s.

(2) $$P_{trans,0}=a\times P_{o:w}{}^{b}$$

Basolateral *P_trans,_*_0_ was manually adjusted to 6000 × 10^−6^ cm/s to cover first pass metabolism and AUC. P-gp CL_int,T_ (μL/min) was calculated by the software from J_max_ of 93 and K_m_ of 9.4, which were collected from the literature [23]. P_para_ was set as 0.05506 × 10^−6^ cm/s, which was the default value in the simulator.

In Simcyp, the gastrointestinal tract is divided into eight sections, including one section for duodenum, two sections for jejunum, four sections for ileum and one section for colon. To better reflect reginal absorption along the GI tract, absorption rate scalars for colon, ileum and jejunum were adjusted in turn to recover PK profiles of locally dosed budesonide solutions. To simulate the administration to corresponding GI sections, ‘Fluid MRT’ and ‘Transit Time (Total%)’ were adjusted accordingly. In the case of colon dosing, as the minimum transit time for small intestine in Simcyp is 0.5 h, the 0.5 h was added to the time axis of the colon-dosed PK profile to compensate for the discrepancy in time course. The absorption rate scalar for duodenum was adjusted to recover the PK profile of orally dosed budesonide solution. As the formulation of locally dosed solution is ethanol/water (1:1), in which the ethanol could substantially increase the permeability [24,25], locally fitted absorption rate scalars across the whole GI tract were adjusted by a cofactor of 0.6 to offset the increase. The final set of absorption scalars used in the model for the duodenum, jejunum, ileum and colon were 0.06, 0.12, 0.54 and 1.44, respectively.

2. Formulation

Controlled/modified release-dispersible system was selected in Simcyp as the formulation type, which tracks the entity of fluid and dissolved drug and also the Pellets with activated Segregated transit time model (STTM) function. Stomach lag time and mean residence time (MRT) of pellet in stomach was set to 0 h and 0.8 h, respectively. MRT of pellet in SI was set to 3 h based on previous clinical investigation on the movement of the particle [13]. Retention time of fluid and dissolved drug was left as default values in the software.

**Figure 2:**
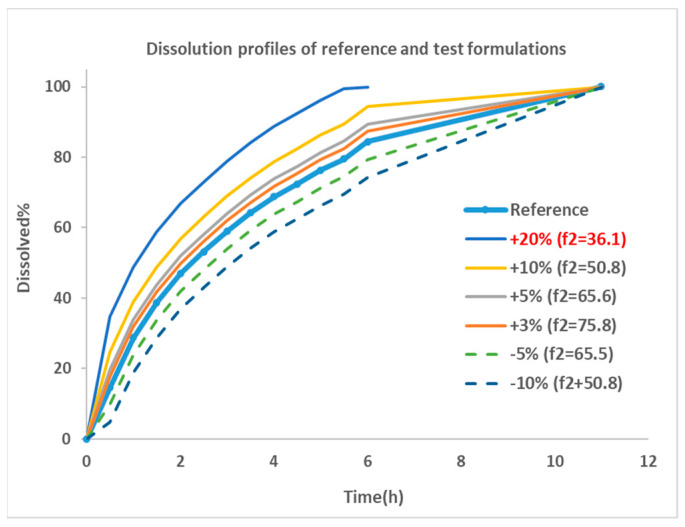
Dissolution profiles for the reference and virtual test formulations. Blue thick line with dot: dissolution profile of reference formulation collected from reference [22]; thin line: formulations showing faster dissolution rate compared with reference; dotted line: formulations with slower dissolution rate; +20%: formulation with 20% more dissolution than reference at every sampling time point, and so on; *f*_2:_ similarity factor.

The dissolution profile for Entocort^®^ EC was collected from reference [22] and used as the dissolution behavior of budesonide pellets. As the dissolution percentage in the experiment did not reach 100% at the last incubation time point, 100% dissolution was assumed at 12 h. Then, the dissolution profile was modified manually by adjusting the % release or altering the trigger pH to generate dissolution profiles for eight virtual formulations. These formulations were named by corresponding changes in dissolution profiles, i.e., ‘+5%’ is the formulations with trigger pH of 5.5 (the same as Entocort^®^ EC) and a 5% increase in the release% at every sampling time point; ‘pH threshold = 5’ is the formulation with a modified trigger pH of 5 and an unchanged release profile compared with reference formulation Entocort^®^ EC. To facilitate subsequent *f*_2_ calculation, the time for achieving 100% dissolution was standardized to 12 h for all formulations except for the +20% formulation, for which 100% dissolution was reached at 7 h. Dissolution profiles for the reference and six virtual formulations modified by changing the release% are shown in Figure 2.

Similarity factor (*f*_2_) between the reference formulation and each virtual formulation were calculated using the bootstrap method [26]. The parameter is a logarithmic reciprocal square root transformation of the sum of squared error and is a measurement of the similarity in the percent (%) dissolution between the two curves, as illustrated in Equation (3).

(3) $$f_{2}=50\times log\left(\frac{100}{\sqrt{1+\frac{\sum_{t=1}^{n}{\left(R_{t}-T_{t}\right)}^{2}}{n}}}\right)$$

where *n* is the number of sampling time points, *R_t_* is the dissolution percentage of reference product at time point t, *T_t_* is the dissolution of test product at time point t. *f*_2_ values greater than 50 are indicative of the sameness or equivalence of the two dissolution curves and, thus, of the performance of the test and reference products [27]. Based on the equation, an *f*_2_ of 50 could be achieved by a formulation with 10% change at every time point of the dissolution profile compared with the reference formulation. The smaller the change, the greater the *f*_2_ value, until up to 100.

Dissolution profiles for reference and six virtual formulations were then fitted to the Weibull function using Equation (4). Corresponding *F_max_*, α and β for Entocort^®^ EC and virtual formulations are listed in Table 2.

(4) $$F=F_{max}\times (1-\exp\left(\frac{-{\left(t-T_{\mathrm{lag}}\right)}^{\beta }}{\alpha }\right)$$

#### 2.4.5. PBPK Model for Entocort^®^ EC in Crohn’s Disease Patients

3. Local sensitivity analysis (LSA)

As Crohn’s disease could lead to significant anatomical and physiological changes that might alter the pharmacokinetic property of drugs, LSA was conducted to assess the influence of changes in physiological parameters to the PK profiles of Entocort^®^ EC, and to build CD patient population by modifying key parameters. The selection of parameters for sensitivity analysis was based on the properties of budesonide and reported changes in CD patients, including mean retention time (MRT) in gut and small intestine; CYP3A4 abundance in liver, small intestine (SI) and colon; HSA concentration; and transporter abundance. Ranges of these parameters were set to cover the range for both healthy subjects and CD patients. Details are listed in Table 3. Oral C_max_ and AUC_last_ were selected as the analysis endpoints.

4. Demographic parameters for healthy volunteers and Crohn’s disease patients

The default setting for healthy volunteers in the Simcyp database was used in the PBPK model of Entocort^®^ EC in healthy subjects. CD population was built by changing key parameters identified in LSA. Values of these parameters in CD patients were collected from literature. The list of parameters and values are listed in Table 4.

### 2.5. Virtual Bioequivalence (VBE)

The bioequivalence between Entocort^®^ EC and virtual formulations were simulated by VBE module in Simcyp software (version 22). The study design for all simulations was the typical design for BE study, i.e., two-sequence, two-treatment, two-period, crossover study. All simulations were performed with 10 trials with 12 subjects in each trial. Healthy subjects age from 20 to 50, with 50% of female. Virtual subjects were generated from demographic information by the simulator using the Monte Carlo method. The default between-subject variability for HV population built in the software internal database was applied to each parameter. Within-subject variances (WSV) for some parameters were included to better recover the actual variance, as listed in Table 5.

After simulation, concentration–time profiles in the system/plasma and two layers (lumen and enterocyte) of eight sections of GI tract (duodenum, Jejunum 1 and 2, ileum 1–4, and colon) for each subject after each treatment were generated and exported to Excel and then imported to WinNonlin for bioequivalence analysis. PK parameters (AUC_last_ and C_max_) for each subject were calculated by noncompartmental analysis. A linear mixed-effect model was used to analyze the variance in log-transformed PK parameters between formulations. Sequence, treatment and period were selected as fixed effects, and subjects-within-sequence was regarded as random effect.

**Figure 3:**
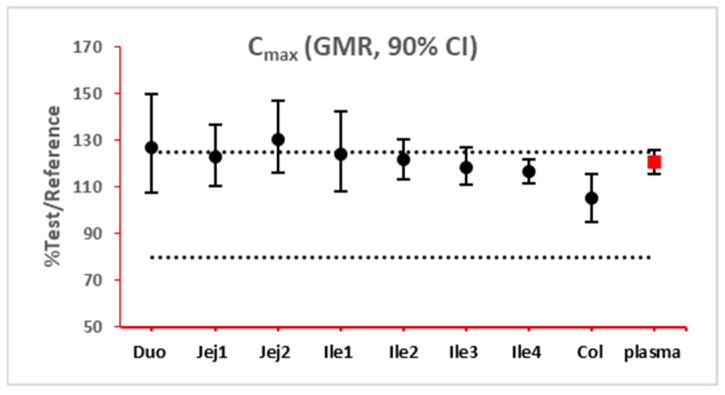
Example bioequivalence bar chart based on C_max_. Gray dotted line: 80% and 125%; black dots: geometric mean of test versus reference in GI segments; red square: geometric mean of test versus reference in plasma; bar: 90% confidence of the geometric mean of test over reference. GMR: geometric mean of test/reference; CI: confidence interval. Duo: duodenum; Jej1 and Jej2: jejunum sections 1 and 2; Ile1, Ile2, Ile3 and Ile4: ileum sections 1 to 4; Col: colon.

The 90% confidence interval (CI) of the ratio of geometric mean (test/reference) of PK parameters was calculated. Two formulations were considered bioequivalent if the 90% CI of the ratio of geometric means of C_max_ and AUC_last_ fell within the bioequivalence limits of 80% to 125%. BE bar charts were prepared based on BE results in the enterocyte or lumen layer of 8 GI sections, and plasma in one trial (Figure 3).

**Figure 4:**
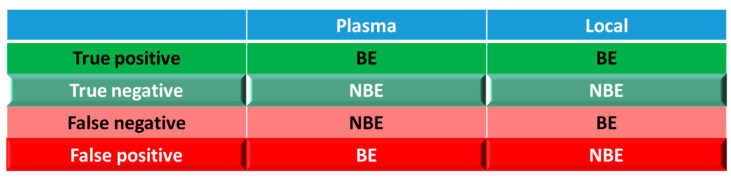
Coloring and naming rule for the heatmap. Positive: bioequivalent; negative: not bioequivalent; true: local GI segment consistent with plasma, colored in green; false: local GI in consistent with plasma, colored in red.

For simulations with 10 trials, if ≥8 trials show bioequivalence, the formulation was defined as bioequivalent to Entocort^®^ EC. BE heatmaps were then generated based on bioequivalence or nonbioequivalence (NBE) results between 8 virtual formulations and Entocort^®^ EC in plasma and the local GI tract in 10 trials (Figure 4). If a formulation was BE with Entocort^®^ EC in plasma, the result was defined as positive; otherwise, the result is negative. If the BE in certain GI segment was consistent with that in plasma, i.e., plasma BE could represent local BE result, then the result was defined as true and colored in green (true positive or true negative); otherwise, the result was considered false and colored in red and pink (false positive or false negative).

## 3. Results

### 3.1. PBPK Models for Entocort^®^ EC in Healthy Volunteers and CD Patients

Detailed parameters for Entocort^®^ EC PBPK models for healthy volunteers are summarized in Table S1. Simulations for both healthy volunteers and CD patients met the criterion for internal (0.8- to 1.25-fold) and external validation (within 2-fold) (Table 6), indicating the success of models in predicting the exposure of budesonide after the administration of solutions and the formulation. Regarding the shape of the simulated concentration–time profile, as shown in Figure S1, disposition and clearance of budesonide was successfully recovered by the PBPK model with IV bolus dose in healthy volunteers. Furthermore, the PK profiles of budesonide after local and oral administration of 2.6 mg (1 mL) solution, 3 mg (10 mL) solution and Entocort^®^ EC were also captured well (Figures S2–S5 and S9). Details about local sensitivity analysis can be found in Figures S7 and S8).

Regarding local absorption in the GI tract, the ileo-colonic region accounts for approximately 70% of absorption% of budesonide, and the simulated value (82.2%) has no significant difference from the reported value (76.1%), as shown in Table S2. The fraction of absorption in each segment was simulated well except for transverse and descending colon (reported: 6.9% vs. simulated: 1.9%).

Simulated concentration–time profiles for different sections and layers of the GI tract were checked visually in Figure S6, and simulated t_max_ and C_max_ were compared, as listed in Table 7. Plasma concentration reached C_max_ at 3 h while the upper small intestine reached C_max_ earlier, which was at 1–2 h for the duodenum and jejunum. The time for the local GI tract to reach C_max_ was longer for distal intestinal sections. T_max_ for the ileum and colon were 2–3 and 6 h, respectively. The shape of the concentration–time profile (in semilog scale) in the colon is quite similar to that in plasma. In contrast, the concentration profile of the duodenum to ileum segments exhibited a completely different shape. It might be caused by the long retention time and thus prolonged absorption of the drug in the colon. For the same section of the gastrointestinal tract, concentrations in lumen and enterocyte showed a consistent trend of changes. Enterocyte concentrations are around 1% of the lumen concentrations in the same segment. Furthermore, concentrations in lumen and enterocytes are 2–5 orders of magnitude higher than the plasma concentration at the same sampling time point.

### 3.2. Virtual BE Heatmaps for Healthy Subjects

Systematic and local BE results were organized into heatmaps for further analysis. To facilitate classification and identification, heatmaps were modified based on local BE results. Local BE cells remained flat, while 3D bevel effects were added to local NBE cells. After modification, local BE could be identified by the 3D effects of cells. Flat green cells represent the most favorable scenario for drug discovery companies, i.e., BE in both plasma and local GI segments. Light green bevel cells depict situations that show systemically and locally NBE. In both green cases, local BE could be expected through comparative PK studies. However, light green is less desirable since it may indicate the failure of the generic drug. Two red colors were used in the heatmap to represent unfavorable situations. Red bevel cells mean systematically BE, but locally NBE. In this case, substandard products could be released through PK-based BE studies and that would be unfavorable for patients. Flat pink cells represented the situation of systematic NBE while achieving BE in the corresponding GI segment, implying that one might lose generic drugs that could potentially work appropriately.

**Figure 5:**
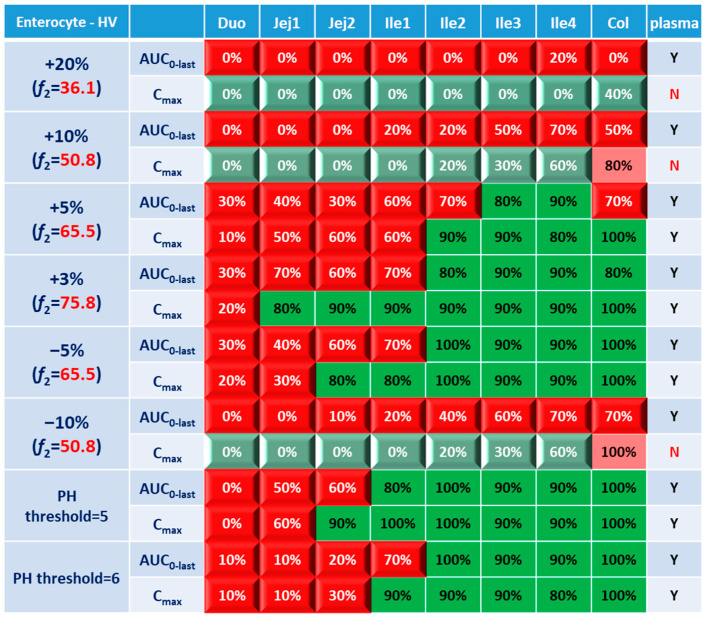
BE heatmap of 8 virtual formulations based on AUC and C_max_ in the enterocyte layer of GI sections and plasma in the healthy population. Percentage in the box indicates the incidence of BE result in 10 trials. Y indicates BE in plasma. N means NBE in plasma. Green: local BE result consistent with that in plasma. Red: local BE result inconsistent with plasma. Cells with 3D bevel effect: not BE in local GI section. Flat cells: BE locally.

BE heatmap for the enterocyte and plasma of the healthy volunteer population is shown in Figure 5. Regarding the sensitivity of the two parameters, i.e., AUC and C_max_, the latter provided more negative results in plasma, whereas AUC identified more negative cases in local GI tracts. Regardless of the parameter used to identify NBE result, it is challenging to identify BE results in upper intestinal sections, including the duodenum and jejunum. It suggests that these regions are sensitive to changes in the dissolution rate and trigger pH, which could be attributed to variations in the formulation manufacturing process.

In Figure 5, BE results based on AUC_0–last_ and C_max_ were listed separately to compare the sensitivity of these two parameters. In plasma, all the test formulations were bioequivalent to the reference formulation based on AUC_0–last_, while three formulations (+20%, +10% and −10%) were identified to be not bioequivalent based on plasma C_max_. It suggests that plasma AUC_0–last_ is less sensitive to formulation changes than plasma C_max_. On the other hand, in the local GI tracts, AUC_0-last_ is more sensitive than C_max_ since the former identified more local NBE results. Due to the discrepancy in parameter sensitivity, it is necessary to combine AUC_0–last_ and C_max_ together when checking the discordance in local and systemic bioequivalence.

**Figure 6:**
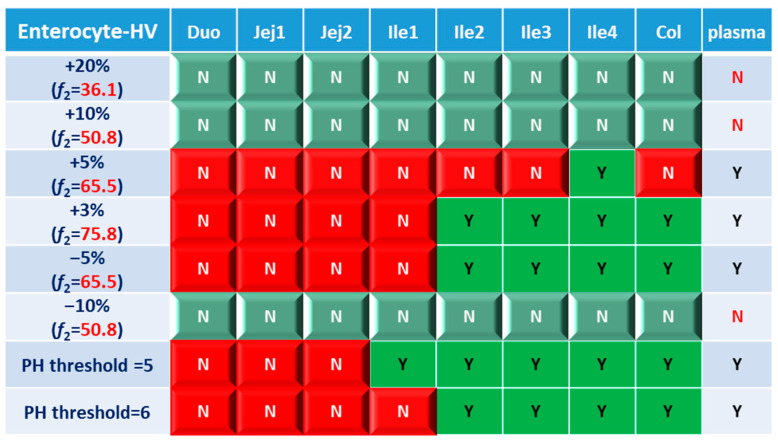
BE heatmap of 8 virtual formulations based on the combined results of AUC and C_max_ in the enterocyte layer of GI sections and plasma in the healthy population. Y indicates BE in GI segments and plasma. N means NBE in GI segments and plasma. Green: local BE result consistent with that in plasma. Red: local BE result inconsistent with plasma. Cells with 3D bevel effect: not BE in local GI section. Flat cells: BE locally.

Based on the combined BE results derived from both AUC and C_max_ (Figure 6), nearly all the formulations show NBE results based in the upper intestines (duodenum and jejunum), which means upper intestines are very sensitive to formulation changes. Both ends of the GI tract (duodenum, jejunum, ileum 1 and colon) were observed to be sensitive to changes in the dissolution rate, and the ileum section tends to be conservative to formulation changes and showed more BE results than other sections. Based on simulation results of eight formulations, in most cases plasma BE could represent the local GI BE in the ileum 2–4 sections and colon, except for the +5% formulation, which was bioequivalent in plasma but not in the ileum 2–3 or colon.

Regarding the performance of similarity factor *f*_2_ in predicting plasma and local BE, the study suggested that the commonly used cutoff of 50 is insufficient to ensure either systemic BE or local GI BE. Bioequivalence of plasma BE could be achieved for formulations when *f*_2_ is increased to 65.5. Regarding bioequivalence in local GI sections, the duodenum, jejunum and ileum 1 segments are so sensitive to formulation changes that all formulations in the current study were found to be not bioequivalent in these sections. Regarding to the more conservative ileum 2–4 and colon sections, which are also the target sections for Entocort^®^ EC, bioequivalence could be achieved when *f*_2_ reaches 65.5, i.e., the +3% formulation (*f*_2_ = 75.8) and −5% formulation (*f*_2_ = 65.5). But for another formulation with *f*_2_ of 65.5, i.e., the +5% formulation, local BE in the ileum 2–3 and colon was not achieved. In summary, based on the BE heatmap, *f*_2_ of 50 is inadequate to ensure local or plasma bioequivalence in the case of the formulation Entercort^®^ EC. Higher *f*_2_ values (>65.5) should be considered as quality control for GI locally acting products.

For two formulations with altered trigger pH, which could be achieved by altered coating material or thickness of coating, BE performance is generally comparable or even superior to that of formulations with high *f*_2_ values (65.5 or 73.8). NBE results were observed in the upper sections (duodenum to ileum 1), whereas the ileum 2–4 and colon were conservative to formulation changes. Increasing the trigger pH from 5.5 to 6.0 could potentially delay the release of the drug after administration, and thus lead to more significant change compared with the performance-to-formulation with a trigger pH of 5.0.

**Figure 7:**
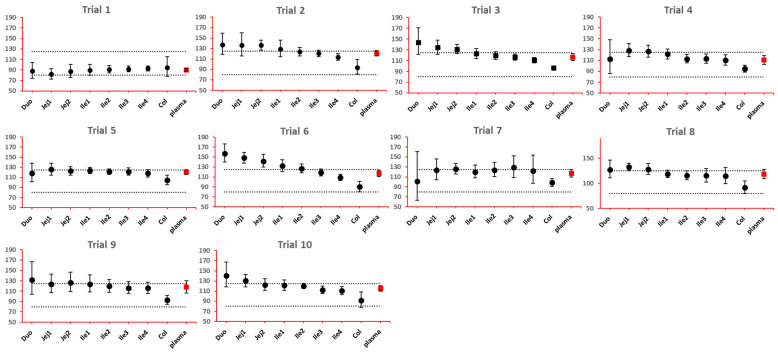
BE bar charts (based on C_max_) of the +10% formulation in 10 trials. Gray dotted line: 80% and 125%; black dots: geometric mean of test versus reference in GI segments; red square: geometric mean of test versus reference in plasma; bar: 90% confidence of the geometric mean of test over reference. GMR: geometric mean of test/reference; CI: confidence interval.

**Figure 8:**
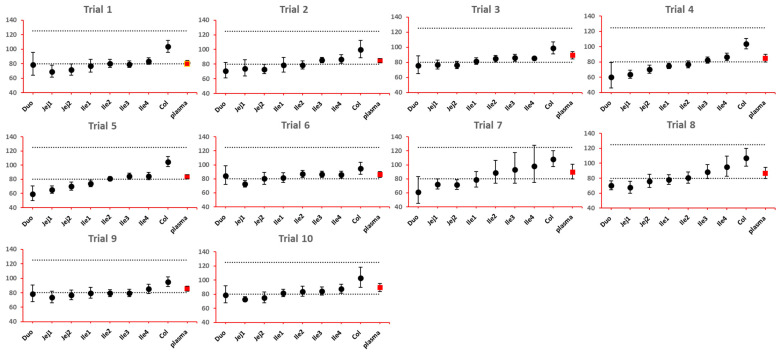
BE bar charts (based on C_max_) of the −10% formulation in 10 trials. Gray dotted line: 80% and 125%; black dots: geometric mean of test versus reference in GI segments; red square: geometric mean of test versus reference in plasma; bar: 90% confidence of the geometric mean of test over reference. GMR: geometric mean of test/reference; CI: confidence interval.

As NBE results could be attributed to the shift in the geometric mean, or wide confidence interval related to high interindividual variance, BE bar charts for two formulations with faster and slower dissolution rates were examined. As depicted in Figure 7 and Figure 8, the 90% confidence interval range in local GI tracts are wider than that in plasma, suggesting higher interindividual interval. However, the interval is not excessively wide to cause negative outcomes in bioequivalence analysis. Regarding the trend of concentration change, a faster dissolution rate leads to higher concentration in the upper gastrointestinal tracts as well as in plasma in all trials; slower dissolution could lead to a lower concentration in the upper gastrointestinal tract as well as in plasma. The concentration in colon tends to move in the opposite direction compared with plasma. The magnitude and direction of concentration change in the ileum sections are generally the same as that in plasma, possibly accounting for synchronization between plasma BE and ileum local BE.

**Figure 9:**
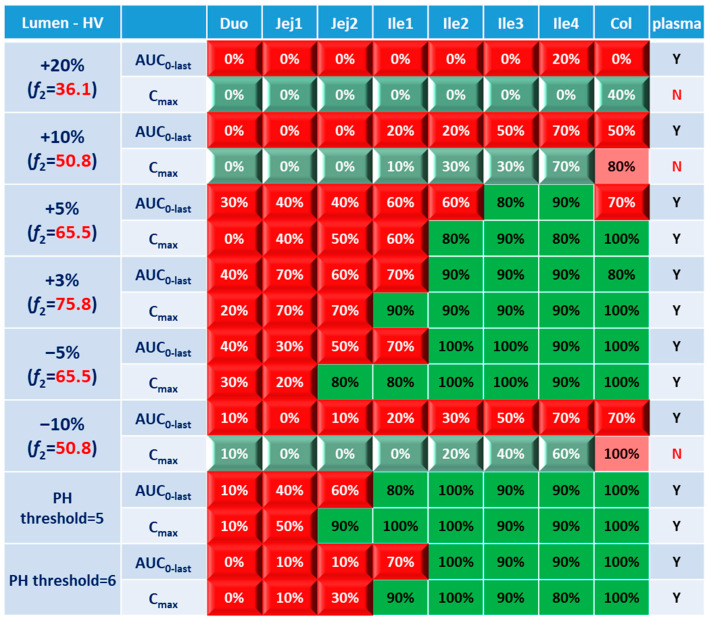
BE heatmap of 8 virtual formulations based on AUC and C_max_ in the lumen layer of GI sections and plasma in the healthy population. Percentage in the box indicates the incidence of BE result in 10 trials. Y indicates BE in plasma. N means NBE in plasma. Green: local BE result consistent with that in plasma. Red: local BE result inconsistent with plasma. Cells with 3D bevel effect: not BE in local GI section. Flat cells: BE locally.

As lumen concentration is important to other GI local action drugs treating Crohn’s disease, such as metronidazole and ciprofloxacin, etc., BE results based on lumen concentrations and PK parameters were also examined for eight formulations and were compared with BE results simulated in plasma. As shown in Figure 9, comparison between the lumen and enterocyte heatmaps did not uncover any significant difference, especially when one considers the sensitive parameter C_max_. Similar phenomenon concerning discrepancy between local and systemic BE and the discrimination effect of *f*_2_ could be observed. Although the concentrations in the lumen layer is around 100 fold that of the enterocyte concentration in same GI section (Table 7), they generally respond to the formulation modifications in the same manner and to the same extent.

### 3.3. Virtual BE Heatmaps for CD Patients

**Figure 10:**
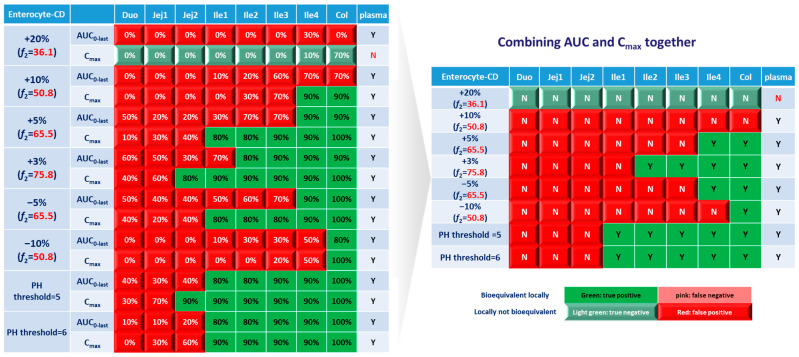
BE heatmap of 8 virtual formulations based on AUC and C_max_ in the enterocyte layer of GI sections and plasma in Crohn’s disease patients. Percentage in the box indicates the incidence of a BE result in 10 trials. Y indicates BE in plasma. N means NBE in plasma. Green: local BE result consistent with that in plasma. Red: local BE result inconsistent with plasma. Cells with 3D bevel effect: not BE in local GI section. Flat cells: BE locally.

The ultimate goal of a bioequivalence study is to achieve the same local concentration profile and thus therapeutic effect in Crohn’s disease patients. Although investigating local and systemic BE in the CD patient population can be challenging, valuable insights can be obtained through the power of PBPK modeling. In this study, BE in local GI tracts and plasma simulated in CD patients based on lumen and enterocyte were examined. As with healthy volunteers, the lumen layer tends to give similar BE results with the enterocyte compartment; a BE heatmap for the enterocyte layer was prepared and depicted in Figure 10.

Similar to the observations in the healthy population, C_max_ tends to yield more negative BE results in plasma, whereas AUC_0-last_ is associated with higher negative rate in local GI tracts. However, for CD patients, almost all virtual formulations showed bioequivalence in plasma. In the systemic BE point of view, *f*_2_ of 50 seems to be a reliable indicator of bioequivalence. Nevertheless, more simulations with formulations having *f*_2_ values lower than 50 need to be conducted to confirm the conclusion.

Regarding sensitivity along the GI tract, as observed in healthy subjects, the upper intestinal tract (duodenum, jejunum and ileum 1) in CD patients was observed to be more sensitive to changes in formulation, compared with plasma and colon. The observations in ileum sections of patients are significantly different from that in healthy populations. For CD patients, the conservative area restricted to ileum 4, where three formulations with *f*_2_ > 65.5 showed local bioequivalence. Most parts of the ileum (ileum 1–3) are sensitive to the formulations. To achieve local bioequivalence in almost the entire ileum, an *f*_2_ higher than 75.8 might be required, which means only a 3% increase at each sampling time point is tolerated in the dissolution profile. As for another important target area, the colon, it seems to be the most conservative GI segment in patients. All three formulations with *f*_2_ > 65.5 (+5%, +3% and −5%) and one formulation with *f*_2_ = 50.8 (−10%) showed bioequivalence in this area.

Concerning formulations with altered trigger pH, it seems that such changes could be well tolerated in the ileum and colon sections of CD patients, since all these segments showed bioequivalence results.

Based on these simulation results on altered dissolution rate and trigger pH, local concentrations in the ileum and colon sections were very sensitive to changes in the dissolution rate of formulations, which could not be reflected by clinical BE study based on PK profiles. To ensure qualified products being provided to patients, attention should be given to carefully characterize the dissolution profile; a product-specific higher *f*_2_ (75.8) might be needed for QC of Entocort^®^ EC to ensure an appropriate BE in CD patients.

## 4. Discussion

This current work describes the development and validation of PBPK models for Entocort^®^ EC, which is a locally acting GI formulation treating Crohn’s disease in the ileum and ascending colon. Model parameters were carefully adjusted to reflect the actual movement of budesonide pellets along the GI tract, as well as regional absorption in local GI sections. The final model successfully recovered regional absorption in the colon, ileum and jejunum, along with the systemic exposure after oral administration. After validation against clinical PK profiles in healthy subjects and CD patients, virtual bioequivalence between Entocort^®^ EC and eight virtual formulations was simulated, and BE heatmaps based on systematic and local exposure were prepared. Through advanced modeling methods, bioequivalence at the site of action and its correlation with the upstream dissolution and downstream system exposure under various scenarios were simulated and explored.

For GI local action drugs with measurable systemic exposure (like Entocort^®^ EC), the quality of products or qualification of generic drugs are controlled by the in vitro dissolution profile and by a clinical BE trial. A minimum *f*_2_ of 50 is required for the dissolution of the new batch or generic drug under investigation [27]. Our simulation results suggested that an *f*_2_ of 50 is not sufficient to ensure systemic BE in healthy volunteers, but it appears to be an appropriate cutoff value to ensure BE in the plasma of CD patients. For the correlation between *f*_2_ and local BE, due to the varied sensitivity of GI sections to formulation change, different *f*_2_ standards should be applied based on the target GI sections. For drugs targeting the ileum and colon sections, an *f*_2_ of 65 seems required to ensure BE in these two sections in healthy volunteers. In contrast, the ileum of CD patients showed very high sensitivity to changes in dissolution rate, and an *f*_2_ of 75 should be met to achieve ileum BE. The colon section in CD patients showed better tolerance to formulation change compared with the ileum, and an *f*_2_ of 50 could relate to a certain possibly of colon BE. Combining observations in the ileum and colon together, an *f*_2_ of 75 might be a better cutoff to ensure local BE in the ileum and colon sections.

Concerning the validity of clinical bioequivalence studies in demonstrating bioequivalence in local GI tracts, our results suggest that these studies may be useful in identifying formulations that are NBE in the whole GI tract, i.e., formulations show NBE result in systemic circulation are probably NBE in all gastrointestinal segments. However, on the other hand, formulations that show BE results based on systemic exposure could be BE or NBE in GI tracts, depending on the location of the segment of intestine that is of interest. Thus, a clinical BE result should only be the minimum standard for products that tend to be BE in GI segments.

The major pitfall for the current simulation is that, based on the product specific guidance of Entocort^®^ EC, in vitro dissolution studies should cover a series of pH values including pH 4.5 in citric acid and pHs 6.0, 6.5, 6.8, 7.2 and 7.5 in PBS [29]. In contrast, the dissolution profiles for budesonide pellets used in the model were collected with FaSSIF medium. The calculated *f*_2_ for virtual formulations under investigation might be different if the dissolution profiles were collected according to product-specific guidance. But we believe the dissolution profile in FaSSIF could better mimic the actual dissolution behavior of budesonide pellets in the gastrointestinal tract. Moreover, establishing BE in the case of drugs where a metabolite contributes to pharmacological or safety aspects requires further considerations beyond what we have demonstrated here.

## 5. Conclusions

Despite such shortcomings for generalization, our results provide a proof-of-concept that local BE does not necessarily parallel systemic BE in the case of drug/formulations acting locally in the GIT. Establishing local BE remains a challenging area that probably requires full and complete clinical studies with pharmacological endpoints in target patients. Our work also suggests that virtual bioequivalence studies conducted with appropriate PBPK models could be used to decide on local BE effectively.

## Figures and Tables

**Table 1:** Summary of clinical studies used for model development (1–4) and validation (5–10) in healthy subjects and validation (11, 12) in CD patients.

No.	Formulation	Dose	No. of Subjects (Gender) ^a^	Age	Weight (Kg)	Reference
1	IV	0.5 mg	12 (M); 12 (F)	22–53	45–92	[10]
2	Solution (local)	2.6 mg (1 mL)	8 (M)	20–44	63–111	[11]
3	Solution	3 mg (10 mL)	6 (M); 6 (F)	43.7 ± 7.1	71.5 ± 10.3	[12]
4	Entocort^®^ EC	18 mg	8 (M)	40–53	77–94	[13]
5	Entocort^®^ EC	3, 9, 15 mg	5 (M); 8 (F)	NA	NA	[14]
6	Entocort^®^ EC	4.5 mg	6 (M)	43–56	NA	[15]
7	Entocort^®^ EC	9 mg	6 (M); 6 (F)	21–42	NA	[16]
8	Entocort^®^ EC	3 mg	8 (M)	22–40	85 (66–107)	[17]
9	Entocort^®^ EC	4.5 mg	40 (F)	19–38	61.5 (46–86)	[18]
10	Entocort^®^ EC	3 mg	8 (M)	20–42	75 (60–91)	[19]
11	Entocort^®^ EC	9 mg	4 (M); 4 (F)	24–50	BMI 24.9 (18.5–29.7)	[20]
12	Entocort^®^ EC	1 mg	1 (M); 7 (F)	25–70	57.4–104	[21]

^a^ M: male; F: female.

**Table 2:** Similarity factor and Weibull function parameters for Entocort^®^ EC and virtual formulations.

Formulation	*f*_2_	Similarity between R and T	F_max_	α	β	Trigger pH
Entocort^®^ EC	-	-	100	3.12	0.94	5.5
+20%	36.1	N	100	1.53	0.87	5.5
+10%	50.8	Y	100	2.14	0.89	5.5
+5%	65.5	Y	100	2.56	0.91	5.5
+3%	75.8	Y	100	2.77	0.92	5.5
−5%	65.5	Y	100	3.90	1.01	5.5
−10%	50.8	Y	100	5.01	1.09	5.5
pH threshold = 5	-	-	100	3.12	0.94	5
pH threshold = 6	-	-	100	3.12	0.94	6

**Table 3:** Parameters included in LSA and corresponding ranges.

Parameters	Ranges Covered by LSA	Range in HV	Reported Ranges in CD Patients		
			[22]	[23]	[28]
Gastric MRT (h)	0.27–2.5	0.27	0–2.5	0.26 (Active); 0.3 (Inactive)	
SI MRT (h)	3.4–6	3.4	3–6	4.2 (Active); 3.2 (Inactive)	
Liver CYP3A4 abundance (pmol/mg protein)	34.35–137	137	31.5 (M), 45.75 (F) (low); 38.49 (M), 55.91 (F) (high)	55.4 (M); 80.5 (F)	
SI CYP3A4 abundance (nmol/SI)	8.6–65.4	65.4	60.53 (low); 98.53 (high)	52.3	8.6 (Inflamed); 15.6 (Noninflamed)
HSA (g/L)	30–50	50.34 (M); 49.38 (F)	31.72 (M), 27.2 (F) (low); 41 (high)	Study one: 30.13 (M); 25.2 (F) Study two: 44.8 (M); 43.9 (F)	
Colon CYP3A4 abundance (nmol/colon)	0.2–1.99	1.99		2.4	0.2 (Inflamed); 0.5 (Noninflamed)
Transporter abundance (pmol/mg total membrane protein)	Jejunum I: 0.075–0.4	Jejunum I: 0.4		Ileum I–IV:1.2 Colon: 0.17 (Active); 0.55 (Inactive)	Jejunum I: 0.12 (Inflamed); 0.075 (Noninflamed)

M: male; F: female; MRT: mean residence time; SI: small intestine; HSA: human serum albumin. HV: healthy volunteers; CD: Crohn’s disease. High: Crohn’s disease high level population reflecting the high level for each parameter. Low: Crohn’s disease low level population reflecting the low level for each parameter. Active: Crohn’s disease is active and lead to symptoms like pain, diarrhea or fatigue. Inactive: Crohn’s disease is in remission and no more symptoms. Inflamed: Inflamed intestinal segments taken from active CD patients undergoing ileocolonic resection. Noninflamed: histologically normal intestinal segments taken from active CD patients undergoing ileocolonic resection.

**Table 4:** Demographic parameters that were adjusted to build the CD population.

Parameter	HV	CD	Reference
Liver CYP3A4 abundance (pmol/mg protein)	137	55.4 (M); 80.5 (F)	[23]
SI CYP3A4 abundance (nmol/SI)	65.4	8.6	[28]
Colon CYP3A4 abundance (nmol/colon)	1.99	0.2	[28]
HSA (g/L)	50.34 (M); 49.38 (F)	30.13 (M); 25.2 (F)	[23]

Note: HV: healthy volunteers. CD: Crohn’s disease patients. HV data were default settings in Simcyp database.

**Table 5:** Within-subject variability used in VBE studies of Entocort^®^ EC.

Parameters	Variation (CV%)	Minimum Limit	Parameter Value	Maximum Limit
Fasted MRT Stomach Fluid	38.217	0.01	0.27	12
Fasted MRT SI Fluid	21.132	0.5	3.4	12
Male WColon MRT Fluid	44.962	0.1	37.5	240
Male AColon MRT Fluid	44.962	0.1	18.91	72
Female WColon MRT Fluid	44.962	0.1	55.75	240
Female AColon MRT Fluid	44.962	0.1	23.11	72

MRT: mean residence time; WColon: whole colon; AColon: ascending colon.

**Table 6:** Overview of predicted and observed PK parameters and calculated fold error.

Clinical Study	Subject	Formulation	Dose (mg)	Observed Values		Simulated Values		Ratio: sim/obs	
				AUC_0–t_ (nM × h)	C_max_ (nM)	AUC_0-t_ (nM × h)	C_max_ (nM)	AUC_0–t_	C_max_
1	HV	IV bolus	0.5	15.27	11.1	12.66	9.12	0.83	0.82
2-1	HV	Solution (Jejunum) *	2.6 (1 mL)	8.52	3.14	8.19	3.38	0.96	1.08
2-2	HV	Solution (Ileum) *	2.6 (1 mL)	11.77	5.31	11.18	5.19	0.95	0.98
2-3	HV	Solution (Colon) *	2.6 (1 mL)	8.56	2.36	10.05	2.44	1.17	1.03
3	HV	Solution (Oral)	3 (10 mL)	6.58	2.15	8.30	1.84	1.26	0.86
4	HV	Entocort^®^ EC	18	51.49	5.92	54.78	5.74	1.06	0.97
5-1	HV	Entocort^®^ EC	3	12.98	1.77	9.17	0.96	0.71	0.54
5-2	HV	Entocort^®^ EC	9	38.65	3.74	27.68	2.95	0.72	0.79
5-3	HV	Entocort^®^ EC	15	59.37	7.08	45.83	4.81	0.77	0.68
6	HV	Entocort^®^ EC	4.5	18.72	2.21	14.06	1.47	0.75	0.67
7	HV	Entocort^®^ EC	9	26.41	4.18	21.47	2.80	0.81	0.67
8	HV	Entocort^®^ EC	3	12.24	1.16	8.23	0.87	0.67	0.75
9	HV	Entocort^®^ EC	4.5	13.15	1.39	14.03	1.45	1.07	1.04
10	HV	Entocort^®^ EC	3	11.75	1.28	8.23	0.88	0.70	0.69
11	CD patients	Entocort^®^ EC	9	27.27	4.32	45.35	5.39	1.66	1.25
12	CD patients	Entocort^®^ EC	1	4.41	0.56	4.35	0.52	0.99	0.93

* Simulated with regional absorption scalars before applying a coefficient of 0.6.

**Table 7:** Simulated t_max_ and C_max_ in plasma and different sections of the GI tract.

Endpoint	t_max_ (h) Lumen/Enterocyte	C_max,lumen_ (nM)	C_max,enterocyte_ (nM)
Plasma	3	0.94	-
Duodenum	1/0.5	459	2.67
Jejunum I	1/1	10,455	43.2
Jejunum II	2/2	16,745	31.3
Ileum I	2/2	20,767	102
Ileum II	3/2	17,769	91.1
Ileum III	3/3	17,825	83.3
Ileum IV	3/3	15,935	75.2
Colon	6/6	152,267	1092

## Data Availability

The data can be shared up on request.

## Supplementary Materials

The following supporting information can be downloaded at: https://www.mdpi.com/article/10.3390/pharmaceutics15092237/s1, Model verification using systemic and locally measured concentration profiles.
