# Supplementary material for: Understanding Discordance between In Vitro Dissolution, Local Gut and Systemic Bioequivalence of Budesonide in Healthy and Crohn’s Disease Patients through PBPK Modeling
Source: Pharmaceutics. 2023 Aug 30;15(9):2237. doi: 10.3390/pharmaceutics15092237 (PMC10535222; doi:10.3390/pharmaceutics15092237)
Supplement: Supplementary file 1 [file pharmaceutics-15-02237-s001.zip › pharmaceutics-2563823-supplementary.pdf]

Understanding Discordance between In Vitro Dissolution, Local Gut and Systemic Bioequivalence of Budesonide in Healthy and Crohn's Disease Patients through PBPK Modeling

Chunyan Han [1,2,*], Tiancheng Sun [2], Siri Kalyan Chirumamilla [3], Frederic Y. Bois [3], Mandy Xu [2] and Amin Rostami-Hodjegan [1,3,*]

[1] Centre for Applied Pharmacokinetic Research (CAPKR), University of Manchester, Manchester M13 9PL, UK
[2] Pharmaron Inc., Beijing 100176, China; tiancheng.sun@pharmaron.com (T.S.); mandy.xu@pharmaron.com (M.X.)
[3] Simcyp Division, Certara, Sheffield S1 2BJ, UK; sirikalyan.chirumamilla@certara.com (S.K.C.); frederic.bois@certara.com (F.Y.B.)
* Correspondence: chunyan.han@pharmaron.com (C.H.); amin.rostami@manchester.ac.uk (A.R.-H.); Tel.: +86-010-5791-5816 (C.H.)

Supplementary File. Model Verification Using Systemic and Locally Measured Concentration Profiles

Table S1: Parameters used in budesonide PBPK model

Parameter	Value	Reference/Comments
**Phys chem and blood binding**		
Compound type	neutral	
Molecular weight (g/mol)	430.5	
Log $P_{o:w}$	2.62	[1]
f_u	0.15	
B:P	0.8	
Plasma Binding Component	HSA	[2]
K_D to HSA (μM)	118.53	Calculated by Simcyp based on fu of 0.15
Intrinsic solubility (μg/mL)	0.028	[2]
**Distribution**		
Model	Full PBPK, Method2	
K_p scalar	0.8	Calculated based on observed volume of distribution in IV PK profile
**Elimination**		
CYP3A4 CL_{int} (μL/min/10^6 cell)	4.1	Fitted value
CL_R typical renal clearance for a 20-30yr healthy male(L/h)	1.55	Calculated by fu and the GFR of population representative
**Absorption**		
Model	Multi-layer gut wall within ADAM (M-ADAM) model	
Apical $P_{trans,0}$ (all segments)(10^{-6}cm/s)	1783	Calculated by Simcyp by method 2 based on LogP
Basolateral $P_{trans,0}$ (all segments) (10^{-6}cm/s)	6000	Manually adjusted to recover AUC
P_{para} (10^{-6}cm/s)	0.05506	default
Absorption rate scalars	Duodenum: 0.06<br>Jejunum I-II: 0.12<br>Ileum I-IV: 0.54<br>Colon: 1.44	Fitted from observed data of locally-administrated budesonide solution
Paracellular Effective Molecular Radius	7.5508	default
P-gp $CL_{int,T}$ (μL/min)	42.35	Estimated based on best fit to observed PO data of budesonide solution
Use GI volume accessible	On	

Parameter	Value	Reference/Comments
surface area		
Capillary bed permeability-surface area product (L/h)	40	default
Effective Concentrations	Free aqueous concentration	
$D_{eff,bul}$ Scaler	1	default
Formulation	Controlled/modified release-dispersible system	
Dissolution profile	Weibull	
F_{max}	100	
Alpha	3.1196	Fitted from in-vitro dissolution test data
Beta	0.93998	
Trigger PH	5.5	
Use segregated transit time model	on	
Permit MRT and lag time of particles and pellets to be less than that of fluid	on	
Pellet lag time in stomach (h)	0	
Pellet mean residence time (h)	Stomach: 0.8<br>Small intestine: 3	[3]

S1. PBPK Models for Healthy Subjects

S1.1. IV administration

The performance of the PBPK model in recovering the disposition and clearance of budesonide was assessed by the simulation of PK profile after intravenous bolus dose of 0.5 mg budesonide. Budesonide disposition was successfully simulated as shown in Figure S1. Predicted PK parameters were within the predefined 0.8- to 1.25-fold range for internal model verification.

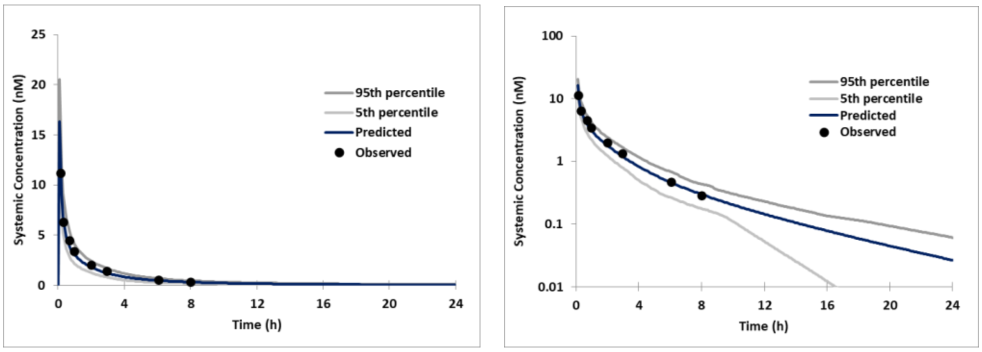

(a) (b)

Figure S1. Simulation of budesonide plasma concentration for healthy subjects after intravenous administration of 0.5mg budesonide. (a) normal scale; (b) right, semi-log scale.

S1.2. Solution and Entocort® EC

The exposure of budesonide after local and oral administration was simulated by PBPK model of 2.6 mg (1 mL) solution and 3 mg (10 mL) solution, respectively. After that, the exposure of orally-given budesonide was simulated by model of 18 mg Entocort® EC. The model for extended-release capsule was then externally validated against observed PK data collected in 8 clinical studies with different doses of Entocort® EC.

Regarding the locally-administrated PBPK model, all the ratio of parameters fell within a 0.8-1.25-fold range and concentration time profiles were recovered well by visually check (Table S2, Figure S2). For the orally-administrated solution PBPK model, AUC was overpredicted (1.26-fold) and slightly beyond range of 0.8-1.25 for internal validation. (Table S2, Figure S3) Then the in vitro dissolution profile of the extended-release formulation was incorporated in the oral model to build the PBPK model for Entocort® EC. Simulated AUC and C_{max} in the internal validation with 18 mg Entocort® EC were within the predefined 0.8- to 1.25-fold range (Table S2, figure S4). External validation against 8 clinical PK studies (Table S2, Figures S5) showed that AUC and C_{max} were all recovered well (within 2-fold of reported values).

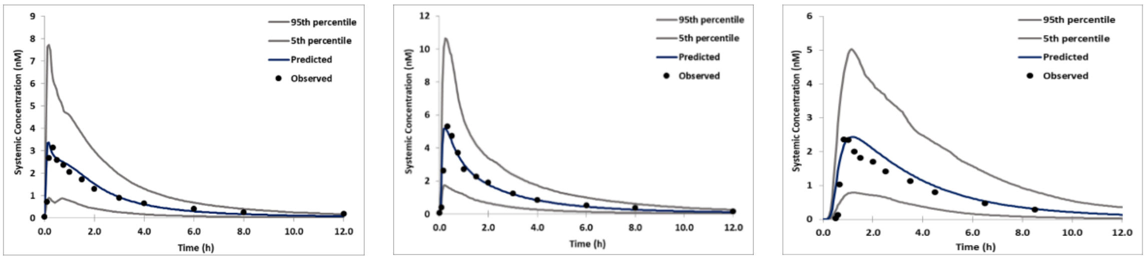

(a) (b) (c)

Figure S2. Simulation of budesonide plasma concentration for healthy subjects after local administration

of 2.6 mg budesonide solution (a) Jejunum; (b) Ileum; (c) Colon.

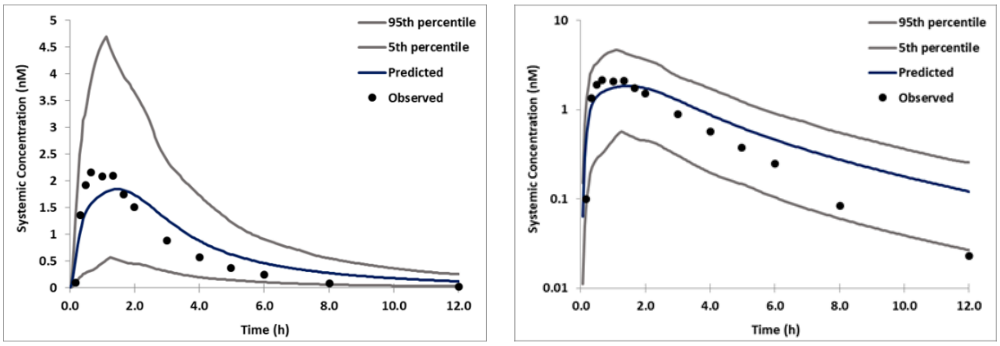

(a) (b)

Figure S3. Simulation of budesonide plasma concentration for healthy subjects after oral administration of 3 mg budesonide solution. (a) normal scale; (b) semi-log scale.

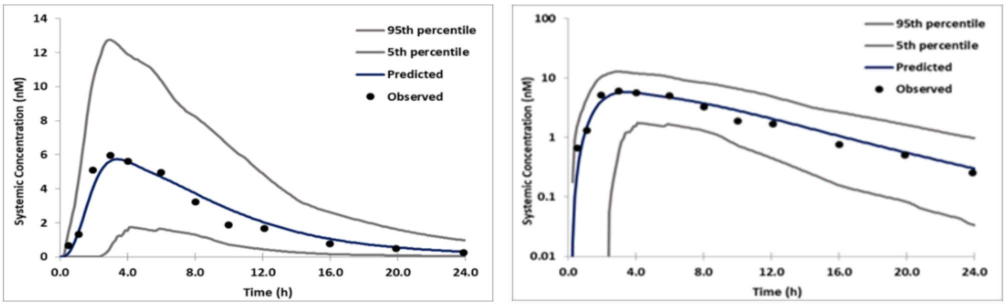

(a) (b)

Figure S4. Simulation of budesonide plasma concentration for healthy subjects after oral administration of Entocort® EC containing 18 mg budesonide. (a) normal scale; (b) semi-log scale.

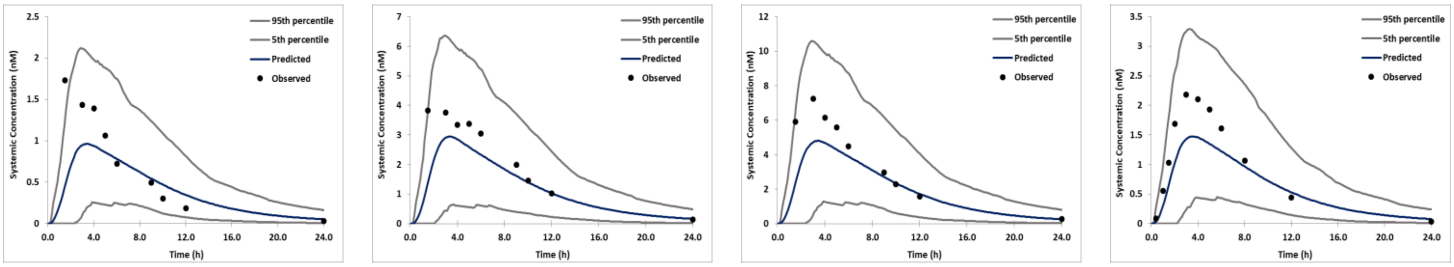

(a) (b) (c) (d)

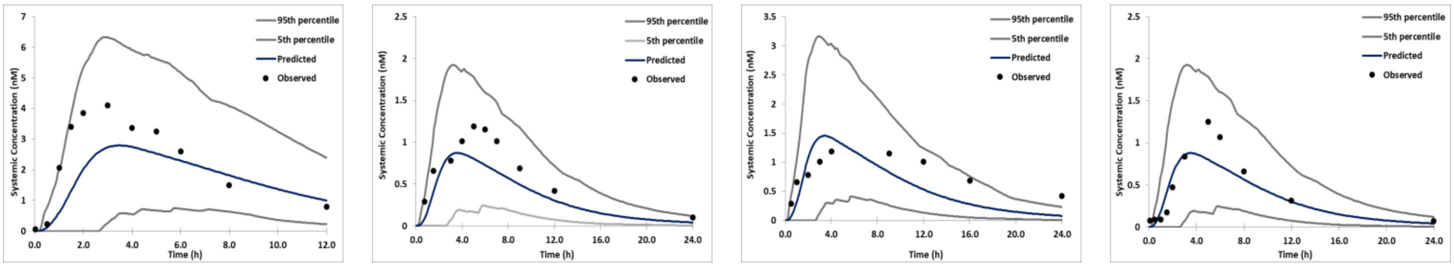

(e) (f) (g) (h)

Figure S5. Simulation of budesonide plasma concentration for healthy subjects after oral administration of Entocort® EC with different doses for external validation. (a) Study 5-1; (b) Study 5-2; (c) Study 5-3; (d) Study 6; (e). Study 7; (f) Study 8; (g) Study 9; (h) Study 10.

S1.3. Absorption fraction in GI tract

Table S2. Simulated and observed % absorption [3] in local GI tract by deconvolution.

Section	Healthy volunteers		CD patients	
	**Reported mean (95% CI)**	**Simulated**	**Reported mean (95% CI)**	**Simulated**
**Upper small intestine**	24.4 (11.8, 37.0)	17.9	32.0 (17.4, 46.6)	26.8
**Ileum**	26.6 (19.6, 33.6)	28.4	17.0 (10.9, 23.1)	16.1
**Ascending colon**	42.0 (30.8, 52.7)	51.9	25.3 (12.2, 38.5)	49.4
**Transverse + descending colon**	6.9 (0.5, 14.3)	1.9	26.2 (6.6, 45.8)	7.7
**Total**	100.5	100.1	100.5	100

S1.4. Simulations of GI tract local concentrations

As bioequivalence analysis in different sections and layers of GI tract, and the investigation of potential difference between local bioequivalence and plasma bioequivalence were the purpose of this research, simulated concentration-time profiles in these layers/sections in GI tract was checked visually in Figure S6 and simulated t_{max} and C_{max} were compared as listed in Table S7. For jejunum and ileum which have multiple sequential compartments in Simcyp, only the first compartment was included in the figure.

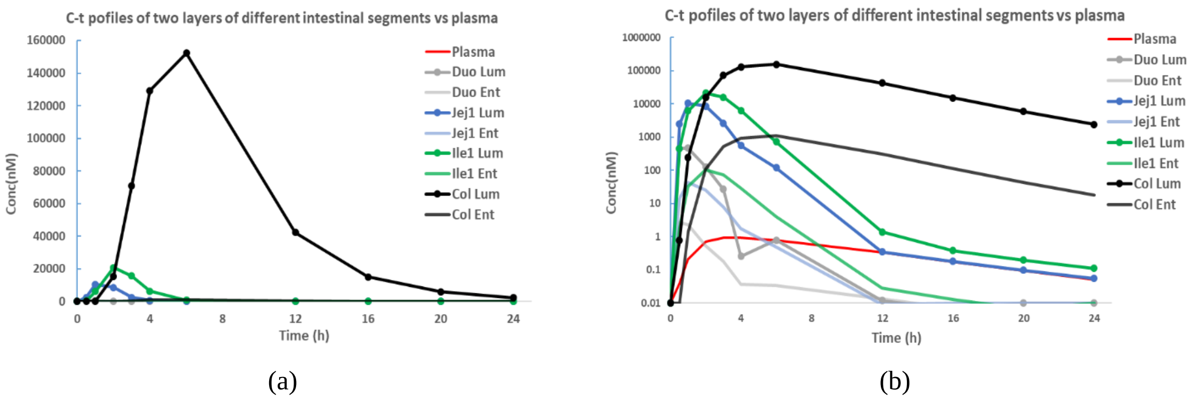

Figure S6. Observed plasma concentration (red dots), simulated plasma concentration (red line) and simulated concentrations (lines) in lumen and enterocyte layers of duodenum (grey), jejunum 1 (blue), ileum 1 (green) and colon (black). (a) normal scale; (b) semi-log scale.

S1.5. Local sensitivity analysis

To figure out the most relevant pathophysiological changes in CD patients resulting potential alternations in AUC and C_{max} of budesonides after dosing of Entocort® EC, seven parameters were included in local sensitivity analysis. As illustrated in Figure S7 and S8, liver CYP3A4 abundance, SI CYP3A4 abundance, colon CYP3A4 abundance and HSA concentration could influence the pharmacokinetics of budesonide substantially, whereas retention time in stomach and small intestine, and P-gp expression level showed minor effects. Among 4 major effects, hepatic CYP3A4

abundance showed the most significant influence. An enzyme reduction of 76% (137 pmol/mg protein in healthy subjects to 34.35 pmol/mg protein in CD patients) could result in a 3-fold increase of both AUC and C_{max}. It is easy to understand since the hepatic extraction ratio of budesonide is around 0.6, and CYP3A4 accounts for the majority of hepatic metabolism. The second most influential parameter is SI CYP3A4 abundance. The change from 65.4 nmol/SI in healthy subjects to 8.6 nmol/SI in CD patients could lead to potential increase of AUC and C_{max} by 19nM*h and 4.1nM, which account for 52% and 90%, respectively. HSA concentration also determines the pharmacokinetics of budesonide. A decrease by 25 g/L (50.34 g/L in healthy population to 25.2 g/L in CD patients) resulted in an ~50% decrease in both AUC and C_{max}. A 10-fold difference of colon CYP3A4 abundance resulted in an 83% increase of AUC but only 20% for C_{max}.

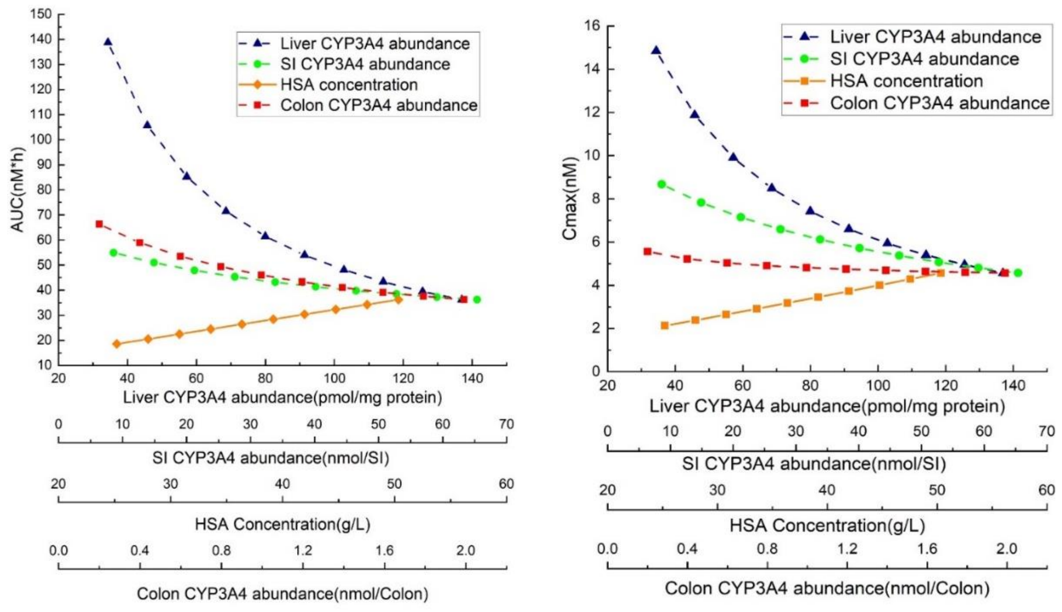

(a) (b)

Figure S7. Local sensitivity analysis of the parameter which showed significant effects on AUC(a) and C_{max}(b)

Although GI tract transit time could determine how long the drug is exposed to the digestive tract and thus the absorption percentage, gastric MRT and SI MRT showed very low impact on the exposure of budesonide. Even though budesonide is a P-gp substrate, the decrease of transporter abundance from 0.4 to 0.075 pmol/mg total membrane protein didn't lead to any noticeable change in AUC and C_{max}. Further examination revealed a minor increase (1%) in Fa, as well as a minor decrease (0.5%) in Fg along with the decrease in transporter abundance. The combined result of these two processes was that AUC and C_{max} remained almost constant. .

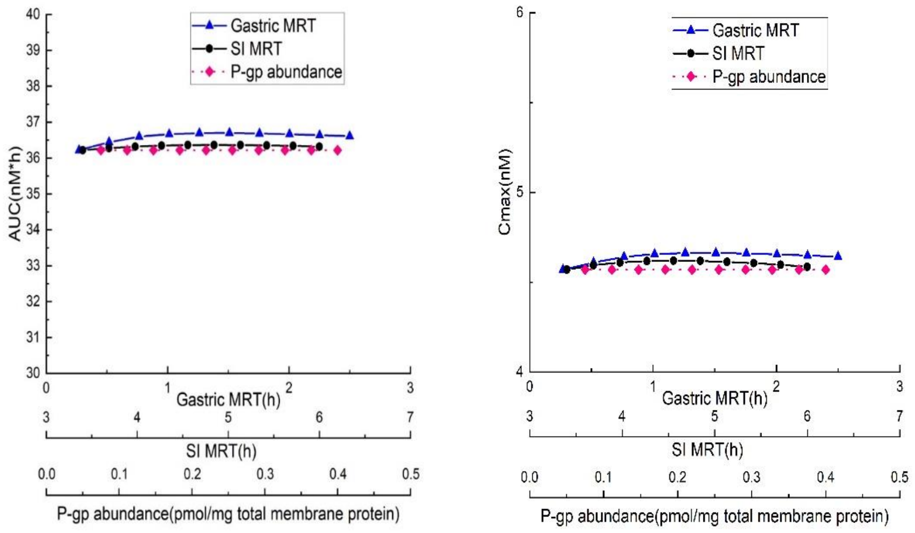

(a) (b)

Figure S8. Local sensitivity analysis of the parameter which showed minor effects on AUC (a) and C_{max} (b)

S2 Model for Crohn's Disease Population

Based on the result of sensitivity analysis, parameters that show major influence to pharmacokinetic behavior of budesonide were modified to build CD patient population. Predicted and observed budesonide plasma profiles after Entocort® EC (containing 9 mg of budesonide) administration in CD patients in fasted state are shown in Figure S9. The respective PK parameters are presented in Table S2. Results suggest that the PK profile of budesonide could be appropriately simulated by the PBPK model, and simulated AUC and C_{max} are both within the 2-fold range for external validation.

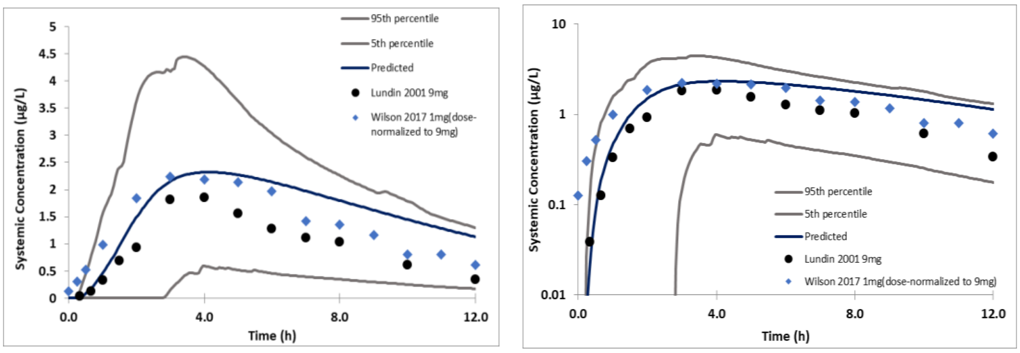

(a) (b)

Figure S9. Simulation of budesonide plasma concentration for CD patients after oral administration of Entocort® EC with 9 mg budesonide. (a) normal scale; (b) semi-log scale.

Although the fitting parameters (simulated/observed) of the Entocort® EC PBPK model in CD patients passed the 2-fold cut-off, clearance of budesonide in CD patient seems to be underestimated as indicated by the shape of the terminal elimination phase. Further investigation showed that the slope in this terminal phase is greatly influenced by the colon CYP3A4 abundance. Increasing the

colon CYP content from 0.2 in CD patients to higher values could better recover the observed patient PK profiles while having almost no influence to C_{max}. As the reported colon CYP3A4 abundance was collected with intestinal tissues collected in surgery, indicating certain seriousness of the disease, the current model was used in subsequent BE analysis representing a worst-case scenario.

References

1. Effinger, A.; O'Driscoll, C. M.; McAllister, M.; Fotaki, N. Predicting budesonide performance in healthy subjects and patients with Crohn's disease using biorelevant in vitro dissolution testing and PBPK modeling. *European Journal of Pharmaceutical Sciences* **2021,** *157*, 105617.
2. Alrubia, S.; Mao, J.; Chen, Y.; Barber, J.; Rostami-Hodjegan, A. Altered bioavailability and pharmacokinetics in Crohn's disease: capturing systems parameters for PBPK to assist with predicting the fate of orally administered drugs. *Clinical Pharmacokinetics* **2022,** *61*, 1365-1392.
3. Edsbäcker, S.; Bengtsson, B.; Larsson, P.; Lundin, P.; Nilsson, Å.; Ulmius, J.; Wollmer, P. A pharmacoscintigraphic evaluation of oral budesonide given as controlled-release (Entocort) capsules. *Alimentary Pharmacology & Therapeutics* **2003,** *17(4)*, 525-536.
